# Influence of the Physical Training on Muscle Function and Walking Distance in Symptomatic Peripheral Arterial Disease in Elderly

**DOI:** 10.1155/2018/1937527

**Published:** 2018-09-23

**Authors:** Katarzyna Kropielnicka, Wioletta Dziubek, Katarzyna Bulińska, Małgorzata Stefańska, Joanna Wojcieszczyk-Latos, Ryszard Jasiński, Urszula Pilch, Grażyna Dąbrowska, Katarzyna Skórkowska-Telichowska, Dariusz Kałka, Agnieszka Janus, Katarzyna Zywar, Rafał Paszkowski, Anna Rachwalik, Marek Woźniewski, Andrzej Szuba

**Affiliations:** ^1^Faculty of Physiotherapy, University School of Physical Education, al. I. J. Paderewskiego 35, 51-612 Wroclaw, Poland; ^2^WROVASC–an Integrated Cardiovascular Centre, Specialist District Hospital in Wroclaw, Centre for Research and Development, ul. H. Kamińskiego 73a, 51-124 Wroclaw, Poland; ^3^Medical University of Wroclaw, Department of Pathophysiology, ul. Marcinkowskiego 1, 50-368 Wroclaw, Poland; ^4^Wrocław Medical University Department of Internal Medicine, Occupational Diseases and Hypertension, ul. Borowska 213, 50-556 Wroclaw, Poland; ^5^Specialist District Hospital in Wroclaw, Department of Angiology, ul. Kamieńskiego 73a, 51-124 Wroclaw, Poland; ^6^4th Military Clinical Hospital with a Polyclinic in Wroclaw, Department of Internal Medicine, ul. Weigla 5, 50-981 Wroclaw, Poland; ^7^Medical University of Wroclaw, Division of Angiology, Bartla 5 Str., 51-618 Wroclaw, Poland

## Abstract

**Introduction:**

A typical symptom of chronic lower-limb ischaemia is lower-limb pain, which occurs during walking forcing the patient to stop, intermittent claudication (IC). Exercise rehabilitation is the basic form of treatment for these patients.

**Aim:**

The aim of this study was to compare the effectiveness of three types of physical training programmes conducted over a 12-week period in patients with chronic lower-limb arterial insufficiency.

**Materials and Methods:**

Ninety-five people qualified for the 3-month supervised motor rehabilitation programme, conducted three times a week. The respondents were assigned to three types of rehabilitation programmes using a pseudo-randomization method: Group I (TW), subjects undertaking treadmill walking training; Group II (NW), subjects undertaking Nordic walking training; Group III (RES+NW), subjects undertaking resistance and Nordic walking training. Treadmill test, 6 Minute Walk Test (6MWT), and isokinetic test were repeated after 3 months of rehabilitation, which 80 people completed.

**Results:**

Combined training (RES+NW) is more effective than Nordic walking alone and supervised treadmill training alone for improving ankle force-velocity parameters (p<0.05) in patients with intermittent claudication. Each of the proposed exercise rehabilitation programmes increased walking distance of patients with intermittent claudication (p<0.05), especially in 6MWT (p=0.001). Significant relationships of force-velocity parameters are observed in the maximum distance obtained in 6MWT, both in Group III (RES + NW) and in Group II (NW) at the level of moderate and strong correlation strength, which indicates that if the lower limbs are stronger the walking distance achieved in 6MWT is longer.

**Conclusions:**

Given both the force-velocity parameters and the covered distance, the training RES + NW gives the most beneficial changes compared to training TW alone and NW alone. All types of training increased walking distance, which is an important aspect of the everyday functioning of people with IC.

## 1. Introduction

Atherosclerotic arterial occlusive disease is the most common cause of peripheral arterial disease (PAD) and lower-limb ischaemia. As a result of the narrowing/occlusion of leg arteries and consequently skeletal muscle hypoxia, the muscles' ability to work decreases [1, 2]. One of the most common symptoms of PAD is intermittent claudication, which manifests itself in lower extremity muscle pain during walking, which subsides after short rest [3]. Repeated episodes of muscle pain contribute to the lowering of physical activity of people with PAD, contributing to disease progression. The chronic ischaemic process leads to a significant reduction in muscle strength, which is most often the result of muscle atrophy and metabolic changes in muscle fibres. In patients with progressive ischaemic disease of the lower limbs, structural changes in skeletal muscles develop due to the process of denervation, myocyte depletion, and selective loss of type II fibres against type I fibres, along with a reduction in the number of motor units [1, 4, 5].

Physical exercise is considered the most important component of the comprehensive treatment process in patients with chronic lower-limb ischaemia, as confirmed in many clinical trials [6–17].

According to the ACC/AHA (American College of Cardiology/American Heart Association) guidelines for the treatment of patients with intermittent claudication, a rehabilitation programme lasting three to six months for 30–45 minutes at a time three times a week is recommended [18, 19]. It is also emphasized that walking training is an integral part of the conservative treatment in combination with prophylaxis and pharmacotherapy.

If conservative treatment does not bring the desired results, endovascular or surgical revascularization is carried out [20].

The purpose of physical training in patients with PAD is to enhance muscle strength, extend the distance of claudication, improve exercise tolerance, haemodynamic parameters, neuromuscular coordination, and quality of life, and postpone surgical treatment [3, 21–23]. At present, the gold standard of rehabilitation for patients with intermittent claudication is supervised walking training on a treadmill. The TASC II (*Trans-Atlantic Inter-Society Consensus II, 2007*) recommendations propose a programme in which both the treadmill angle and speed should be suitably adapted to the patient's abilities, so that between the third and fifth minute of walking, pain of medium intensity occurs (pain intensity according to the ESC scale where 1 means no pain and 5 is maximum pain intensity) [18].

Recently, there have been many scientific publications on Nordic walking training, although it has not been mentioned in recommendations for the rehabilitation process of this group of patients [24]. Briefly, this form of movement is walking with the use of poles, based on a natural human gait. The advantage of Nordic walking training over other aerobic forms (marching, jogging) is higher oxygen consumption and energy expenditure with a lower level of perceived fatigue [25–27]. Upper- and lower-body parts are involved, improving 70–90% of the entire body's muscles [28]. Nordic walking is a simple and easily accessible physical activity, recommended for people of all ages, mainly due to the low risk of falling and injury.

It has been confirmed that regular Nordic walking training in the group of people with PAD influences the claudication distance by extending it [29–32], it improves exercise tolerance [30, 33], and it reduces ischaemic pain [30, 31, 33].

Another type of training dedicated to patients with intermittent claudication is resistance training. Strengthening the muscles of the lower limbs slows down the degenerative processes resulting from chronic ischaemia, as confirmed in studies by McDermott et al. [34]. It turns out that systematically undertaken resistance training increases the volume of muscle fibres of both type I and type II, increases the density of capillaries and muscle strength in general, and also extends the distance of claudication [35–37]. Despite the beneficial effects of resistance exercise, they still attract little attention, both in clinical practices and in scientific research regarding PAD issues.

At each and every stage of the rehabilitation process, physical exercises do bring unquestionable health benefits, in both a somatic and mental capacity. One of the most frequently achieved ambulatory rehabilitation outcomes in patients with intermittent claudication is the extension of claudication distance, which is of great importance in these patients' daily living. However, the exact mechanisms responsible for this are still not fully understood [8, 38].

*Study Aim*. The aim of this study was to compare the effectiveness of three types of physical training programmes conducted over a 12-week period in patients with chronic lower-limb arterial insufficiency.

## 2. Materials and Methods

This publication is part of a project called “WROVASC–An Integrated Cardiovascular Centre”, which was cofinanced by the European Regional Development Fund (POIG.01.01.02-02-001/08-03), within the Operational Programme Innovative Economy, from 2007 to 2013. The study was carried out at the Research and Development Centre of the Regional Specialist Hospital in Wroclaw.

The study was approved by the Ethics Committee of the Medical University of Wroclaw, Poland (Ref. KB-130/2008).

### 2.1. Subjects

Recruitment for the exercise rehabilitation program was carried out at healthcare facilities in Wroclaw during “White Saturdays” on which the patients had free access to consultations with angiologists. The information about “White Saturdays” was promoted by local newspapers, TV, radio, posters, and leaflets.

Over one thousand people were examined, of which 545 reported vascular symptoms. Peripheral vascular disorders (by ABI and Doppler ultrasound) were diagnosed in 219 people. The qualification to the exercise training was continued by Department of Rehabilitation. 144 persons of 219 qualified have agreed to participate in the exercise rehabilitation program.

Inclusion criteria for the exercise training were as follows: over 50 years of age, documented PAD, and lower-limb ischaemia and intermittent claudication distance of 30-400 meters (Fontaine's classification IIa and IIb) stable for at least 3 months, ABI<0.9, sound clinical condition of the patient, and written consent of the patient to participate in the project.

The exclusion criteria included PAD Fontaine stage I (painless distance, no impairment of walking capacity), stage II with walking distance > 400 m and Fontaine stage III/IV (pain at rest / trophic ulcer), uncontrolled arterial hypertension and/or diabetes, decompensated congestive heart failure, level of subjective fatigue above 7 points according to the 10-point Borg scale, cardiovascular incidents (MI, stroke) in the last year prior to the rehabilitation program, revascularization procedures performed during the last 3 months, generally poor patient health, incapacity to perform functional tests in motor terms, mental illness, and participation in another scientific research program.

The patients were qualified by angiologists, cardiologists, and physiotherapists.

**Figure 1:**
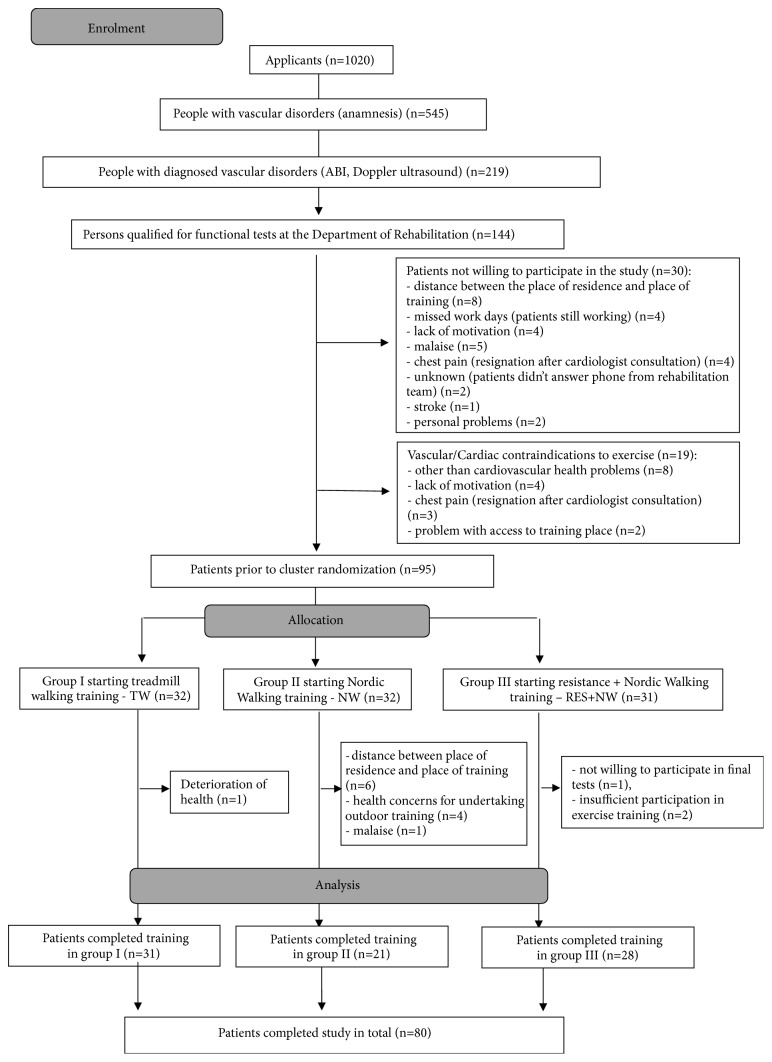
A detailed process of patient recruitment for the rehabilitation programme.

A detailed process of patient recruitment for the rehabilitation program is presented in Figure 1. Finally, 95 people qualified for the 3-month supervised exercise rehabilitation programme, conducted three times a week. The respondents were assigned to three types of training using a pseudo-randomization method.

- Group I (TW): subjects undertaking treadmill walking training
- Group II (NW): subjects undertaking Nordic walking training
- Group III (RES+NW): subjects undertaking resistance and Nordic walking training

Clinical and functional examinations were repeated after 3 months of rehabilitation; 80 people completed the training programs and final testing (Figure 1).

Functional tests, the results of which are presented in this paper, were carried out in the Department of Functional Studies of the Physiotherapy Department at the University of Physical Education in Wroclaw with the assistance of a cardiologist. Characteristics of patients qualified for the rehabilitation programme are presented in Table 1.

### 2.2. Functional Tests

#### 2.2.1. Stress Test on a Treadmill

The test was conducted on a treadmill with a 12-lead electrocardiogram (ECG) and a blood pressure (BP) reading. A Gardner–Skinner protocol was used for the tests, in which belt speed was constant at 3.2 km/h (2.0 mph) and the angle of inclination changed by 2% every 2 minutes. The exercise test was carried out until a maximum claudication pain was reached, fatigue or shortness of breath was reported, or if heart disorders or other disturbing symptoms occurred. Heart rate (HR) and BP measurements were taken before and after each exercise test. The test was performed twice during the morning hours with electrocardiography monitoring in a period of one week. Initial distance of claudication and maximal distance were included in the analysis.

#### 2.2.2. 6-Minute Walk Test (6 MWT)

A 6-minute walk test was conducted in accordance with the American Thoracic Society (ATS) Statement recommendations on a marked 30-meter corridor. The test consisted of the patient's walking at a comfortable pace that he or she generally use on a daily basis. In the situation of maximum pain, which forced the patient to stop during the test, the measured time was not halted. Expressed in meters, the result of the test consisted of the distance of claudication and maximal distance. In this study maximal distance was analysed. The degree of subjective level of fatigue was assessed according to Borg's 10-degree scale. Before the test, subjects were informed that they could rest during the test in a standing or sitting position, if they experienced intensifying symptoms of exercise intolerance. If during the test severe symptoms of exercise intolerance occurred, which did not disappear despite a temporary rest, the test was immediately stopped. Intensifying symptoms of exercise intolerance that could interrupt the test included shortness of breath, dizziness, blurred vision, sudden sweating, cyanosis, tinnitus, loss of verbal control, general weakness, and fatigue. Before and after the 6MWT test, BP and HR measurements were taken using an automatic sphygmomanometer [39].

#### 2.2.3. A Study of Force-Velocity Parameters of Flexor and Extensor Muscles in the Knee and Ankle Joints

The study was carried out in order to objectively assess the strength of flexor and extensor muscles of the knee joint as well as dorsal and plantar flexor muscles of the ankle joint using a functional dynamometer (Biodex System 4 Pro).

The seat, dynamometer, and a suitable attachment were adjusted so that the tip of the dynamometer became an extension of the axis of rotation in the examined joint. For all respondents the same range of flexion and extension at 90° was determined with an adjustment of the force of gravity. The thigh and pelvis of the respondent were stabilized using straps attached to the chair, so as to eliminate movements in neighboring joints. The starting position was a maximum bending of the lower limb in the investigated joint. The appropriate test protocol was selected, in isokinetic conditions for concentric work.

Before starting the actual measurement, the subject performed three submaximal flexions and extensions and one maximum movement in order to become familiar with a given load. The following loads were applied: for the knee joint, respectively, 180, 60, and 120°/s were used, while for the ankle joint 60 and 120°/s were used. In each test, the respondent performed five alternating limb flexions and extensions in a given joint. It was imperative for participants to exert maximum muscle force in the shortest possible time for each movement. There was a 60-second break between subsequent attempts. During the test, muscle function parameters were recorded: peak torque (PT), total work (TW), and estimated average power (AP).

#### 2.2.4. Training Methods

Physical rehabilitation took place in a 3-month cycle, 3 times a week (36 training units) with a 45-minute duration.

Patients participated in one of three types of training.

(i) Treadmill walking training in Group I (TW) was carried out in accordance with the TASC II recommendations. The patient walked on a moving belt on the HX-100 treadmill with a constant speed of 3.2 km/h at 12° angle of inclination. The training took place in line with interval training principles. The duration of walking was determined by the onset of submaximum claudication pain (level 4 according to the 5-level ACSM scale), after which the patient rested on the nonmoving side strips of the treadmill until the pain subsided (level 0 or 1 according to the ACSM scale). However, the rest period did not exceed 2 minutes. Extending the distance covered was done gradually: at the start the patient covered four sections with breaks for a rest and withdrawal of pain. As the walking capacity improved, the training was extended so that within 45 minutes the patient could walk the longest distance possible. After each section the patient monitored their HR using sensors built into the treadmill grips. The training was individualized and was run concurrently on two treadmills (2 people exercising at the same time).

(ii) Nordic walking training in Group II (NW) was carried out by experienced NW instructors in accordance with the INWA. At the beginning of the training a general warm-up was performed (up to 10 min) using the NW poles (KV+ Campra Clip), followed by walking training with a NW technique using an interval method according to the same rules as in treadmill training. At the end, stretching and breathing exercises were carried out (5 min). After each section, the patient monitored his or her own HR on the radial or carotid artery. Patients walked around the circumference of a large wheel in outside conditions (park) or in a sports hall (in case of bad weather), which allowed for them to be continuously monitored. The first sessions (6–9 units) were devoted to learning a correct NW technique; the following classes were for improvements. The training was performed in groups (up to 12 people in one group).

(iii) Combined training of resistance + Nordic walking in Group III (RES+NW) was carried out alternately (Mon: NW, Wed: RES, Fri: NW, Mon: RES, etc.). The NW training was conducted according to the same rules as in the training above. Isokinetic training (resistance), i.e., conducted under constant angular velocity, while ensuring constant resistance, was based on biological feedback using functional dynamometry on the Biodex S4 Pro. While performing a given movement, the patient controlled its effect on the computer's monitor (biofeedback). Before starting proper training, the patient performed five repetitions at an initial angular velocity of 60°/s (3 reps for warm-up with submaximal force (~70% Fmax) and two with maximum force, in order to determine the training level). After performing a repetition with maximal strength, 70–80% of the maximum value [Nm] was determined for both muscle groups (plantar and dorsal flexors of the ankle joint, or in the case of patients with high arterial occlusion, training for flexors and extensors of the knee joint in both limbs was introduced). The patient's task was to perform a movement with a force that allowed one to reach a limit set at the beginning of the training (70–80%) by means of visual control (on the monitor), in line with the specificity of the biofeedback training. The patient performed 10 repetitions for a given velocity: 60, 120, 180, 240, and 300 and 300, 240, 180, 120, and 60°/s (according to the pyramid rule), altogether totaling 100 movements per limb. After each velocity the patient rested in the intermediate position of the foot or knee for 1 minute. The rest time was extended in the case of a prolonged recovery from claudication pain. The principle of resistance training took place from the lowest velocity (the highest resistance) through to the highest velocity (low resistance, velocity of movement increases: 300°/s), ending again with a high resistance performed at a low velocity of 60°/s.

The NW training took place in a group, while the resistance training was undergone individually, taking place in the Laboratory of Functional Research of the Faculty of Physiotherapy, University of Physical Education in Wroclaw.

### 2.3. Statistical Methods

The distribution and homogeneity of the variance of all analysed parameters obtained by the subjects in each group were examined. Next, mean values, standard deviation, and median were calculated. In order to compare the results obtained in the first and second tests for parameters with a distribution similar to normal, Student's* t*-test was used, whereas when the distribution was not close to normal the Wilcoxon test was applied.

Between the first and the second measurement, participants took part in one of three types of training. In order to compare the effectiveness of the applied form of training, an analysis of univariate variance was made in the case of an assumption of the normality of distribution and homogeneity of variance being satisfied. In the absence of normality of distribution, the Kruskal–Wallis test was used. In the case of a lack of homogeneity of variance, despite a near-normal distribution, the Welch test was applied. If the applied analysis showed statistical significance, calculations were continued with the Scheffe post hoc test.

Spearman correlation coefficient values were calculated between walking distance parameters and force-velocity parameters calculated for each of the study groups before and after the training cycle.

Statistical analysis was carried out using Statistica 13.1.

## 3. Results

The results presented below show the values of distance parameters responsible for walking efficiency and values of force-velocity parameters, which describe the level of muscle function acting on the knee and ankle joints.

### 3.1. Distance Parameters

**Figure 2:**
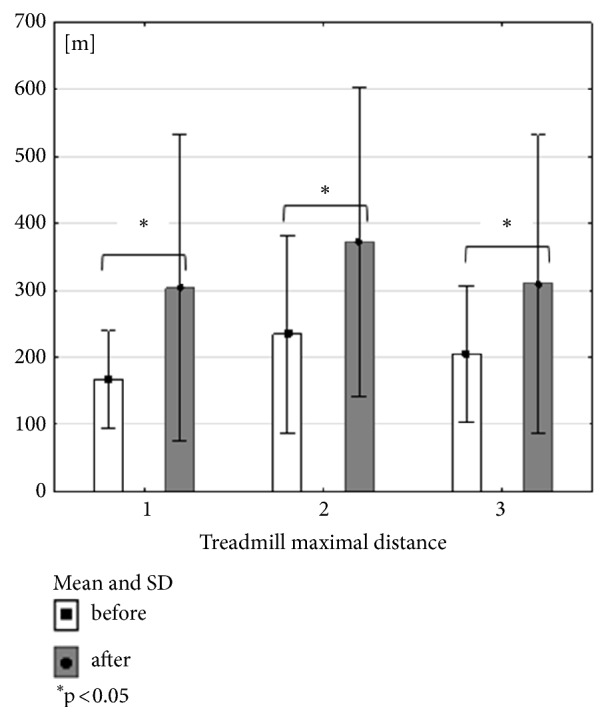
Mean, standard deviation and Wilcoxon coefficient p values calculated between treadmill maximal distance in all groups before and after the training cycle.

**Figure 3:**
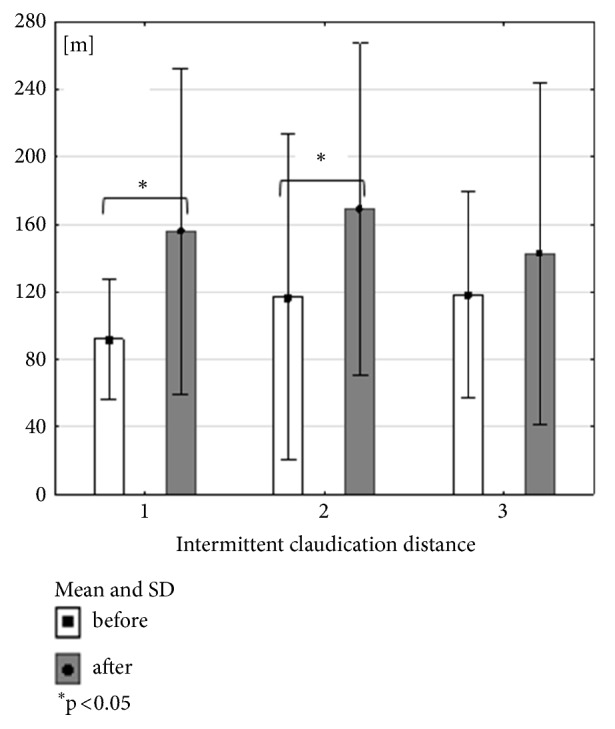
Mean, standard deviation and Wilcoxon coefficient p values calculated between intermittent claudication distance in all groups before and after the training cycle.

**Figure 4:**
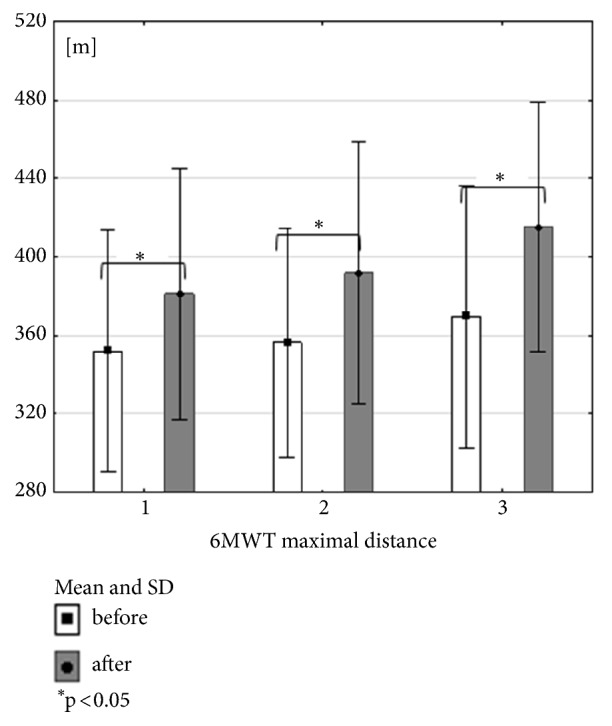
Mean, standard deviation and Wilcoxon coefficient p values calculated between 6MWT maximal distance in all groups before and after the training cycle.

All forms of 3-month exercise training have beneficial influence on maximal distance. Combined training (RES+NW) did not get the significant level in claudication distance. Nordic walking group and treadmill training group had significant influence on claudication distance (Figures 2, 3, and 4). When comparing the groups, no significant differences were recorded, in studies either before or after the training cycle (ANOVA not statistically significant).

### 3.2. Force-Velocity Parameters of the Knee Joint

The results of force-velocity parameters: peak torque (PT [Nm]), total work (TW [J]), and average power (AP [W]) obtained by functional dynamometry tests, typifying the impact of particular types of training on the muscle performance of the knee and ankle joints differently.

As a result of training conducted on the treadmill (Group I), the biggest changes were recorded in the right knee flexors (F) for a velocity of 60°/s and 120°/s in peak torque (PT), total work (TW), and average power (AP) (Table 2).

Nordic walking training improved AP of flexors of the right knee joint for velocities 60°/s and 120°/s and also PT of flexors for a velocity of 120°/s. In the left knee there was only one significant change for extensors in PT parameter for velocity 120°/s (Table 2).

As a result of the combined training (resistance training and Nordic walking) improvement was observed at a velocity of 180°/s in all investigated force-velocity parameters, for both flexors (F) and extensors (E) of the knee joint. There was also changes for knee flexors in both legs at a velocity of 120°/s except one parameter (PT FR) and for knee extensors at this same velocity but observed only in left leg (Table 2).

### 3.3. Force-Velocity Parameters of the Ankle Joint

There were no significant changes recorded in the force-velocity parameters of the dorsal extensor (E) and plantar flexor (F) muscles of the ankle joint in response to the treadmill walking training (Group I) and Nordic walking (Group II) (Table 3).

Combined training (Group III) significantly improved the results in all analysed parameters, for both the dorsal extensor (E) and plantar flexor (F) muscles of the left and right ankle joints at a velocity of 120°/s. At a velocity of 60°/s, this change concerned only the plantar flexors (F) of both ankle joints (Table 3).

### 3.4. Comparison of Results of Force-Velocity Parameters of the Knee and Ankle Joints

In analysing the force-velocity results for both flexors and extensors of both joints (knee and ankle) at different velocities, it turned out that following a 3-month training cycle the most significant changes were observed at a velocity of 180°/s for the knee joint and at 120°/s for the ankle joint. No significant changes were reported at 60°/s for the knee joint (Tables 2 and 3).

### 3.5. Comparison of Results of Force-Velocity Parameters between the Three Forms of Training

Post hoc analysis revealed the most significant changes taking place between the results in Group I (TW) and Group III (RES+NW) at a velocity of 180°/s for the knee joint muscles and 120°/s for the ankle joint muscles. Some significant variations were also observed between Group II (NW) and Group III (RES+NW), with knee joint muscles bearing a velocity load of 180°/s while the ankle joint muscles had a velocity of 60°/s and 120°/s (Tables 4 and 5).

### 3.6. Correlation between Walking Distance and Force-Velocity Parameters

Before the exercise rehabilitation program there were no significant relationships between the maximum distance and the distance of claudication obtained in the treadmill test and force-velocity parameters in all examined groups. The exception is one correlation between the maximum distance and PT ER at 120°/s obtained by Group III. Its value indicates moderate correlation strength (*r*=0.4) (Tables 6 and 7).

The maximum distance obtained in 6MWT before the exercise rehabilitation programme correlates with force-velocity parameters mainly in Group III (RES + NW) for extensors and flexors of the knee joint and extensors of the ankle joint. In Group II (NW) no correlations were noted. In Group I (TW) there were maximal distance compounds with left knee flexors mainly at the speed of 120°/s (Table 8).

After 3 months of exercise training, the most relationships between the maximum distance obtained in the test on the treadmill and the force-velocity parameters are observed in Group III (RES + NW) for flexors and extensors of the knee joint muscles and extensors of the ankle joint (mainly left) (Table 6). In the case of claudication distance, there are single correlations in three exercise groups which have low to moderate strength (Table 7).

The most relationships of force-velocity parameters are observed in the maximum distance obtained in 6MWT, both in Group III (RES + NW) and in Group II (NW) at the level of moderate and strong correlation strength (Table 8). This indicates that if the lower limbs are stronger the walking distance achieved in 6MWT is longer.

## 4. Discussion

A systematically undertaken physical training is the most effective therapeutic treatment for patients with PAD, showing an advantage over pharmacological treatment and angioplasty [16, 40, 41]. It is also the most cost-effective and long-lasting form of treatment for this group of patients [42, 43].

Despite the proven effectiveness of physical training running for at least 3 months, three times a week, a problem that researchers have highlighted is the participation of patients with PAD in these physical rehabilitation programs. Research by De la Hayel et al. [44], Malagoni et al. [45], and Müller-Büh et al. [46] shows that dropout rate from these training programs is as much as 34–44%. Based on the literature review, the main reasons of resignation are not attractive form of exercises (treadmill walking training, recommended by TASC II and ACC / AHA), lack of motivation, health problems, and being far from the place of residence to the centres in which the rehabilitation programs are dedicated for patients with PAD [47]. Despite this, in our study, the dropout rate from the programme was only 16%. One person resigned from training on the treadmill, 11 people from Nordic walking training, and 3 people from combined training (resistance + Nordic walking). The authors observed that outdoor exercises and changing weather conditions were the biggest problem for patients who dropped out from the Nordic walking group, because of deterioration of health status. In the resistance and Nordic walking training this tendency was not found. Impairment of the walking function as a result of chronically occurring claudication pain leads to a significant reduction in the strength and endurance of lower-limb muscles and subsequently to the deterioration of walking skills [1, 48, 49]. This is due not only to the ischaemia of lower-limb muscles but also to the reduced daily physical activity of people with PAD. Introducing resistance training to the standards of therapeutic treatment of people with intermittent claudication seems to be desirable and is confirmed in studies by McDermott et al. [34], McGuigan et al. [35], Parmenter et al. [36], and Wang et al. [37].

Our study suggests that all forms of exercise training have beneficial influence on walking distance. When the force-velocity parameters are taken into account, the combined training (resistance + Nordic walking) caused the most significant changes, comparing to the treadmill training alone and Nordic walking training alone. The most evident effect appears to be the improvement in muscle functions supporting the ankle joint, where peak torque, total work, and average power of left and right lower limbs increased for both the dorsal and plantar flexors at higher velocity of the movement (120°/s). In greater resistance conditions (60°/s), this change occurred only for the flexor muscles of the plantar foot.

Studies by Scott-Okafor et al. [50] confirm that the weakest muscles of the ankle joint in people with intermittent claudication are the dorsal flexors. In comparison to healthy people, the difference in the strength of these muscles is 22% [50]. Furthermore, Chen et al. [51] showed that the torque of dorsal flexor muscles is significantly lower in this group of patients compared to healthy individuals, especially in a situation of developing ischaemic pain. However, what is interesting is that when comparing the torque of both muscle groups in painless and painful walking conditions, it is the flexors of the plantar feet that exhibit significant weakness in response to intermittent claudication [51]. It should be added that the weakness of the muscles supporting the ankle joint can cause an increased risk of falling. Research by Gardner and Montgomery [52] confirms that patients with PAD trip or fall 70% more often than healthy people. It is also important that depending on the speed of the gait the muscles supplying the ankle joint produce 40-60% of the impulsion energy necessary for locomotion [53]. So the resistance training plays significant role in improving walking ability of patients with peripheral arterial disease. In our study, under the impact of combined training, an improvement was achieved in both the dorsal and plantar flexors; however, with greater load, significant change was achieved only in the efficiency of the dorsal flexors of the foot. Presumably, this is due to the physiological superiority of plantar over dorsal flexors in terms of strength; plantar flexors are characterized by a greater muscle mass and number of supplying arteries. Therefore, in this muscle group there is a higher probability of collateral circulation in response to physical training [54], which prevents a reduction in muscle performance of the plantar flexors compared to the smaller and weaker dorsal flexor muscles.

In the literature, the muscles that act on the knee joint have been subjected to a detailed functional analysis under isokinetic conditions, mostly in healthy elderly people and athletes [55, 56]. However, there has been little research on the functional assessment of these muscle groups in patients with chronic diseases [1, 57, 58].

Taking into account changes in the force-velocity parameters of the knee joint, a significant improvement took place mainly in response to the treadmill training and combined training. In the first case, there was improvement only in the right knee flexors at low and medium velocities. This is probably due to the fact that over 60% of respondents had symptoms of intermittent claudication in the right lower limb. In the second case, all analysed force-velocity parameters improved for both flexors and extensors of the knee joint at higher velocity. Studies by McGuigan et al. [35], which showed an increase in the area of muscle fibres types I and II by an average of 25% in response to resistance training, confirm these results. Type I (slow-twitch) fibres are mainly responsible for endurance performance, while type II (fast-twitch) fibres are responsible for high-speed performance, which is why in this study people in Group III performed best with the highest given velocity (180°/s).

One of the most important goals of the rehabilitation of patients with intermittent claudication is to extend their walking distance. In our research, this goal was achieved in response to all the proposed forms of training. Prolongation of the distance of claudication and the maximal distance in response to a 3-month training on a treadmill is unsurprising, given that, as a result of many studies, it has become a recommended form of rehabilitation for patients with intermittent claudication [18]. It should be added that the same form of training, as that of the testing, makes it easier to achieve better results, as observed by Schieb [59].

In recent times, attention has been paid to Nordic walking training by people with PAD, because of similar or even better results being achieved in terms of walking abilities, than training on a treadmill [29–32]. The unconstrained nature of the movement is largely responsible for this, which is similar to the natural walk, in contrast to treadmill training, during which a patient covers the distance at a constant speed, in a nonergonomic position of the body (grabbing the handles for fear of falling). In addition, a great advantage of Nordic walking is training in the open air, providing the patient with more attractions than during the somewhat monotonous training on a treadmill.

In reviewing the literature, it is difficult to find studies which connected endurance and strength training, despite the confirmed benefits of their separate forms, when rehabilitating patients with intermittent claudication. Only in the study by Plitz et al. [60] has it been proposed to combine walking training with prescribed pace, together with strength training performed on the Multiped 303 ergometer. As a result, after 3 months, the speed of walking, the distance of claudication, and the efficiency of the lower-limb muscles assessed in the functional tests all improved [60].

In light of these reports, training that consists of endurance and strength components seems to be a promising form of rehabilitating patients with intermittent claudication. In addition to improvements of the walking distance, which are so important from the daily functioning of patients with PAD point of view, the parameters responsible for strength, endurance, and coordination of lower-limb muscles are also subject to progression. Despite the supervised nature of the combined training, the participants training found it to be attractive with lower drop-out rates comparing to other rehabilitation programs.

## 5. Conclusions

(1) Combined training (resistance and Nordic walking) is the most effective form of exercise training in strengthening the legs and also has beneficial influence on maximal walking distance.

(2) The highest efficiency of combined training (RES+NW), compared to other methods of exercise training, was observed in the force-velocity parameters of the muscles supporting the ankle joint.

(3) Each of the proposed rehabilitation programmes had a positive effect on the walking capacity of patients with intermittent claudication.

(4) The introduction of Nordic walking combined with resistance training should be considered for the therapeutic treatment of patients with intermittent claudication, not only because of the effectiveness of the training, but also in terms of its attractiveness as a form of rehabilitation, preventing dropout from rehabilitation programmes.

## Figures and Tables

**Table 1:** Anthropometric characteristics of patients with PAD and Ankle Brachial Index (ABI).

Variables	Group I	Group II	Group III	p
	(TW)	(NW)	(RES+NW)	
	(n=31)	(n=21)	(n=28)	
	Mean ± SD	Mean ± SD	Mean ± SD	
Age (years)	67.00 ± 7.43	67.00 ± 9.32	67.82 ± 8.49	0.92
Body height (cm)	168.03 ± 8.56	166.81 ± 7.4	169.54 ± 9.07	0.53
Body weight (kg)	79.02 ± 14.31	74.44 ± 12.44	78.84 ± 15.43	0.47
BMI (kg/m^2^)	27.85 ± 3.72	26.77 ± 4.34	27.28 ± 4.01	0.63
ABI R	0.68 ± 0.19	0.76 ± 0.17	0.76 ± 0.19	0.14
ABI L	0.68 ± 0.16	0.71 ± 0.22	0.7 ± 0.19	0.84

*∗* p<0.05; body mass index: BMI and Ankle Brachial Index: ABI.

**Table 2:** *t*-test coefficient values calculated between force-velocity parameters of knee muscles in all groups before and after the training cycle and results of variance analysis calculated between groups for each of the analyzed parameters.

Variables		GROUP I	GROUP II	GROUP III	ANOVA
KNEE		p	p	p	
PT ER	60°/s	0.26	0.28	0.84	ns
TW ER		0.17	0.4	0.58	ns
AP ER		0.07	0.48	0.59	ns
PT EL		0.61	0.17	0.96	ns
TW EL		0.001*∗*	1	0.97	ns
AP EL		0.35	0.13	0.75	ns

PT FR	60°/s	0.03*∗*	0.14	0.31	ns
TW FR		0.02*∗*	0.32	0.46	ns
AP FR		0.01*∗*	0.05*∗*	0.41	ns
PT FL		0.22	0.77	0.68	ns
TW FL		0.22	0.73	0.54	ns
AP FL		0.07	0.72	0.50	ns

PT ER	120°/s	0.16	0.43	0.61	ns
TW ER		0.17	0.81	0.50	ns
AP ER		0.04*∗*	0.29	0.42	ns
PT EL		0.73	0.04*∗*	0.39	0.03*∗*
TW EL		0.50	0.82	0.46	0.01*∗*
AP EL		0.33	0.17	0.41	0.01*∗*

PT FR	120°/s	0.02*∗*	0.02*∗*	0.1	ns
TW FR		0.02*∗*	0.07	0.15	0.03*∗*
AP FR		0.01*∗*	0.01*∗*	0.11	0.03*∗*
PT FL		0.01*∗*	0.56	0.27	0.04*∗*
TW FL		0.09	0.98	0.16	0.02*∗*
AP FL		0.04	0.73	0.17	0.02*∗*

PT ER	180°/s	0.81	0.72	0.3	0.01*∗*
TW ER		0.65	0.1	0.15	0.001*∗*
AP ER		0.36	0.54	0.06	0.001*∗*
PT EL		0.02*∗*	0.44	0.12	0.001*∗*
TW EL		0.08	0.69	0.10	0.001*∗*
AP EL		0.03*∗*	0.52	0.02*∗*	0.001*∗*

PT FR	180°/s	0.54	0.15	0.03*∗*	0.001*∗*
TW FR		0.90	0.52	0.01*∗*	0.001*∗*
AP FR		0.70	0.12	0.00*∗*	0.001*∗*
PT FL		0.02*∗*	0.52	0.05*∗*	0.001*∗*
TW FL		0.09	0.90	0.01*∗*	0.001*∗*
AP FL		0.05*∗*	0.55	0.01*∗*	0.001*∗*

*∗*p<0.05; ns: results statistical not significant, PT [Nm]: peak torque, TW [J]: total work, AP [W]: average power, E: extensor, F: flexor muscles of the knee joint, R: right, and L: left.

**Table 3:** Wilcoxon coefficient p values calculated between force-velocity parameters of ankle muscles in all groups before and after the training cycle and results of variance analysis calculated between groups for each of the analyzed parameters.

Variables		GROUP I	GROUP II	GROUP III	ANOVA
ANKLE		p	p	p	
PT ER	60°/s	0.99	0.53	0.19	ns
TW ER		0.87	0.31	0.11	0.01*∗*
AP ER		0.96	0.31	0.13	ns
PT EL		0.72	0.35	0.22	ns
TW EL		0.72	0.1	0.31	0.01*∗*
AP EL		0.94	0.06	0.23	ns

PT FR	60°/s	0.76	0.47	0.001*∗*	0.001*∗*
TW FR		0.58	0.62	0.001*∗*	0.001*∗*
AP FR		0.54	0.78	0.001*∗*	0.001*∗*
PT FL		0.56	0.49	0.04*∗*	0.001*∗*
TW FL		0.26	0.74	0.02*∗*	0.001*∗*
AP FL		0.31	0.78	0.01*∗*	0.001*∗*

PT ER	120°/s	0.76	0.37	0.01*∗*	0.02*∗*
TW ER		0.8	0.59	0.03*∗*	0.001*∗*
AP ER		0.68	0.91	0.01*∗*	0.001*∗*
PT EL		0.24	0.16	0.01*∗*	0.02*∗*
TW EL		0.54	0.31	0.01*∗*	0.001*∗*
AP EL		0.56	0.29	0.001*∗*	0.001*∗*

PT FR	120°/s	0.8	0.73	0.001*∗*	0.001*∗*
TW FR		0.56	0.43	0.001*∗*	0.001*∗*
AP FR		0.51	0.27	0.001*∗*	0.001*∗*
PT FL		0.53	0.28	0.001*∗*	0.001*∗*
TW FL		0.99	0.27	0.03*∗*	0.001*∗*
AP FL		0.71	0.39	0.001*∗*	0.001*∗*

*∗*p<0.05; ns: results statistical not significant, PT [Nm]: peak torque, TW [J]: total work, AP [W]: average power, E: extensor, F: flexor muscles of the ankle joint, R: right, and L: left.

**Table 4:** Scheffe's post hoc test results showing relationships between results obtained in particular groups (I, TW; II, NW; III, RES + NW) for all analysed parameters of the muscles of the knee joint.

Variables	60°/s			120°/s			180°/s		
KNEE	1vs2	1vs3	2vs3	1vs2	1vs3	2vs3	1vs2	1vs3	2vs3
PT ER	ns	ns	ns	ns	ns	ns	ns	0.01*∗*	ns
TW ER	ns	ns	ns	ns	ns	ns	ns	0.001*∗*	0.01*∗*
AP ER	ns	ns	ns	ns	ns	ns	ns	0.001*∗*	0.02*∗*
PT EL	ns	ns	ns	ns	0.03*∗*	ns	ns	0.001*∗*	ns
TW EL	ns	ns	ns	ns	0.02*∗*	ns	ns	0.001*∗*	0.02*∗*
AP EL	ns	ns	ns	ns	0.01*∗*	ns	ns	0.001*∗*	0.01*∗*

PT FR	ns	ns	ns	ns	ns	ns	ns	0.001*∗*	0.04*∗*
TW FR	ns	ns	ns	ns	0.04*∗*	ns	ns	0.001*∗*	0.001*∗*
AP FR	ns	ns	ns	ns	0.04*∗*	ns	ns	0.001*∗*	0.01*∗*
PT FL	ns	ns	ns	ns	ns	ns	ns	0.001*∗*	ns
TW FL	ns	ns	ns	ns	0.03*∗*	ns	ns	0.001*∗*	ns
AP FL	ns	ns	ns	ns	0.02*∗*	ns	ns	0.001*∗*	ns

*∗* p <0.05; ns: results statistical not significant; PT: peak torque; TW: total work; AP: av. power; E: extension; F: flexion; R: right; L: left.

**Table 5:** Scheffe's post hoc test results showing relationships between results obtained in particular groups (I, TW; II, NW; III, RES + NW) for all analysed parameters of the muscles of the ankle joint.

Variables	60°/s			120°/s		
ANKLE	1vs2	1vs3	2vs3	1vs2	1vs3	2vs3
PT ER	ns	ns	ns	ns	0.03*∗*	ns
TW ER	ns	0.03*∗*	0.03*∗*	ns	0.001*∗*	0.001*∗*
AP ER	ns	ns	ns	ns	0.001*∗*	0.001*∗*
PT EL	ns	ns	ns	ns	0.04*∗*	ns
TW EL	ns	0.04*∗*	0.03*∗*	ns	0.001*∗*	0.001*∗*
AP EL	ns	ns*∗*	ns	ns	0.001*∗*	0.001*∗*

PT FR	ns	0.001*∗*	0.001*∗*	ns	0.001*∗*	0.001*∗*
TW FR	ns	0.001*∗*	0.001*∗*	ns	0.001*∗*	0.001*∗*
AP FR	ns	0.001*∗*	0.001*∗*	ns	0.001*∗*	0.001*∗*
PT FL	ns	0.001*∗*	0.001*∗*	ns	0.001*∗*	0.001*∗*
TW FL	ns	0.001*∗*	0.001*∗*	ns	0.001*∗*	0.001*∗*
AP FL	ns	0.001*∗*	0.001*∗*	ns	0.001*∗*	0.001*∗*

*∗* p <0.05; ns: results statistical not significant; PT: peak torque; TW: total work; AP: av. power; E: extension; F: flexion; R: right; L: left.

**Table 6:** Spearman correlation coefficient values calculated between treadmill maximal distance and force-velocity parameters calculated for each of the study groups before and after the training cycle.

Treadmill maximal distance		BEFORE									AFTER								
		GROUP I			GROUP II			GROUP III			GROUP I			GROUP II			GROUP III		

VS		60°/s	120°/s	180°/s	60°/s	120°/s	180°/s	60°/s	120°/s	180°/s	60°/s	120°/s	180°/s	60°/s	120°/s	180°/s	60°/s	120°/s	180°/s

PT ER	KNEE	ns	ns	ns	ns	ns	ns	ns	0.40*∗*	ns	ns	ns	0.42*∗*	ns	ns	ns	0.51*∗*	0.53*∗*	0.50*∗*
TW ER		ns	ns	ns	ns	ns	ns	ns	ns	ns	ns	ns	0.40*∗*	ns	ns	ns	0.56*∗*	0.50*∗*	0.53*∗*
AP ER		ns	ns	ns	ns	ns	ns	ns	ns	ns	ns	ns	ns	ns	ns	ns	0.57*∗*	0.48*∗*	0.55*∗*
PT EL		ns	ns	ns	ns	ns	ns	ns	ns	ns	ns	ns	ns	ns	ns	ns	ns	ns	0.47*∗*
TW EL		ns	ns	ns	ns	ns	ns	ns	ns	ns	0.38*∗*	0.42*∗*	ns	ns	ns	ns	ns	0.42*∗*	0.49*∗*
AP EL		ns	ns	ns	ns	ns	ns	ns	ns	ns	ns	ns	ns	ns	ns	ns	ns	0.39*∗*	0.50*∗*

PT FR	KNEE	ns	ns	ns	ns	ns	ns	ns	ns	ns	ns	ns	ns	ns	ns	ns	ns	0.41*∗*	0.46*∗*
TW FR		ns	ns	ns	ns	ns	ns	ns	ns	ns	ns	ns	ns	ns	ns	ns	0.42*∗*	0.43*∗*	0.45*∗*
AP FR		ns	ns	ns	ns	ns	ns	ns	ns	ns	ns	ns	ns	ns	ns	ns	0.46*∗*	0.49*∗*	0.43*∗*
PT FL		ns	ns	ns	ns	ns	ns	ns	ns	ns	ns	ns	ns	ns	ns	ns	0.54*∗*	0.42*∗*	0.57*∗*
TW FL		ns	ns	ns	ns	ns	ns	ns	ns	ns	ns	0.54*∗*	ns	ns	ns	ns	0.47*∗*	0.39*∗*	0.53*∗*
AP FL		ns	ns	ns	ns	ns	ns	ns	ns	ns	ns	0.49*∗*	ns	ns	ns	ns	0.42*∗*	0.39*∗*	0.50*∗*

PT ER	ANKLE	ns	ns	-	ns	ns	-	ns	ns	-	ns	ns	-	0.55*∗*	0.46*∗*	-	ns	ns	-
TW ER		ns	ns	-	ns	ns	-	ns	ns	-	ns	ns	-	ns	ns	-	0.45*∗*	ns	-
AP ER		ns	ns	-	ns	ns	-	ns	ns	-	ns	ns	-	ns	ns	-	ns	ns	-
PT EL		ns	ns	-	ns	ns	-	ns	ns	-	ns	ns	-	ns	ns	-	0.56*∗*	0.42*∗*	-
TW EL		ns	ns	-	ns	ns	-	ns	ns	-	ns	ns	-	ns	ns	-	0.57*∗*	0.51*∗*	-
AP EL		ns	ns	-	ns	ns	-	ns	ns	-	ns	ns	-	ns	ns	-	0.51*∗*	0.44*∗*	-

PT FR	ANKLE	ns	ns	-	ns	ns	-	ns	ns	-	ns	ns	-	ns	ns	-	ns	ns	-
TW FR		ns	ns	-	ns	ns	-	ns	ns	-	ns	ns	-	ns	ns	-	0.40*∗*	ns	-
AP FR		ns	ns	-	ns	ns	-	ns	ns	-	ns	ns	-	ns	ns	-	ns	ns	-
PT FL		ns	ns	-	ns	ns	-	ns	ns	-	ns	ns	-	ns	ns	-	ns	ns	-
TW FL		ns	ns	-	ns	ns	-	ns	ns	-	ns	ns	-	ns	ns	-	ns	ns	-
AP FL		ns	ns	-	ns	ns	-	ns	ns	-	ns	ns	-	ns	ns	-	ns	ns	-

*∗* p <0.05; ns: results statistical not significant; -: no measurement.

**Table 7:** Spearman correlation coefficient values calculated between intermittent claudication distance and force-velocity parameters calculated for each of the study groups before and after the training cycle.

Intermittent claudication distance		BEFORE									AFTER								
		GROUP I			GROUP II			GROUP III			GROUP I			GROUP II			GROUP III		
VS		60°/s	120°/s	180°/s	60°/s	120°/s	180°/s	60°/s	120°/s	180°/s	60°/s	120°/s	180°/s	60°/s	120°/s	180°/s	60°/s	120°/s	180°/s

PT ER	KNEE	ns	ns	ns	ns	ns	ns	ns	ns	ns	ns	ns	ns	ns	ns	ns	ns	ns	ns
TW ER		ns	ns	ns	ns	ns	ns	ns	ns	ns	ns	ns	ns	ns	ns	ns	0.38*∗*	ns	ns
AP ER		ns	ns	ns	ns	ns	ns	ns	ns	ns	ns	ns	ns	ns	ns	ns	0.44*∗*	ns	0.38*∗*
PT EL		ns	ns	ns	ns	ns	ns	ns	ns	ns	ns	ns	ns	ns	ns	ns	ns	ns	ns
TW EL		ns	ns	ns	ns	ns	ns	ns	ns	ns	ns	ns	ns	ns	ns	ns	ns	ns	ns
AP EL		ns	ns	ns	ns	ns	ns	ns	ns	ns	ns	ns	ns	ns	ns	ns	ns	ns	ns

PT FR	KNEE	ns	ns	ns	ns	ns	ns	ns	ns	ns	ns	ns	ns	ns	ns	ns	ns	ns	ns
TW FR		ns	ns	ns	ns	ns	ns	ns	ns	ns	ns	ns	ns	0.49*∗*	ns	ns	ns	ns	ns
AP FR		ns	ns	ns	ns	ns	ns	ns	ns	ns	ns	ns	ns	ns	ns	ns	ns	ns	ns
PT FL		ns	ns	ns	ns	ns	ns	ns	ns	ns	ns	ns	ns	ns	ns	ns	ns	ns	ns
TW FL		ns	ns	ns	ns	ns	ns	ns	ns	ns	ns	0.47*∗*	ns	ns	ns	ns	ns	ns	ns
AP FL		ns	ns	ns	ns	ns	ns	ns	ns	ns	ns	0.42*∗*	ns	ns	ns	ns	ns	ns	ns

PT ER	ANKLE	ns	ns	-	ns	ns	-	ns	ns	-	ns	ns	-	ns	ns	-	ns	ns	-
TW ER		ns	ns	-	ns	ns	-	ns	ns	-	ns	ns	-	ns	0.53*∗*	-	ns	ns	-
AP ER		ns	ns	-	ns	ns	-	ns	ns	-	ns	ns	-	0.48*∗*	ns	-	ns	ns	-
PT EL		ns	ns	-	ns	ns	-	ns	ns	-	ns	ns	-	ns	ns	-	ns	ns	-
TW EL		ns	ns	-	ns	ns	-	ns	ns	-	ns	ns	-	ns	ns	-	ns	ns	-
AP EL		ns	ns	-	ns	ns	-	ns	ns	-	ns	ns	-	ns	ns	-	ns	ns	-

PT FR	ANKLE	ns	ns	-	ns	ns	-	ns	ns	-	ns	ns	-	ns	ns	-	ns	ns	-
TW FR		ns	ns	-	ns	ns	-	ns	ns	-	ns	ns	-	ns	ns	-	ns	ns	-
AP FR		ns	ns	-	ns	ns	-	ns	ns	-	ns	ns	-	ns	ns	-	ns	ns	-
PT FL		ns	ns	-	ns	ns	-	ns	ns	-	ns	ns	-	ns	ns	-	ns	ns	-
TW FL		ns	ns	-	ns	ns	-	ns	ns	-	ns	ns	-	ns	ns	-	ns	ns	-
AP FL		ns	ns	-	ns	ns	-	ns	ns	-	ns	ns	-	ns	ns	-	ns	ns	-

*∗* p <0.05; ns: results statistical not significant; -: no measurement.

**Table 8:** Spearman correlation coefficient values calculated between 6MWT maximal distance and force-velocity parameters calculated for each of the study groups before and after the training cycle.

6MWT maximal distance		BEFORE									AFTER								
		GROUP I			GROUP II			GROUP III			GROUP I			GROUP II			GROUP III		
VS		60°/s	120°/s	180°/s	60°/s	120°/s	180°/s	60°/s	120°/s	180°/s	60°/s	120°/s	180°/s	60°/s	120°/s	180°/s	60°/s	120°/s	180°/s

PT ER	KNEE	ns	ns	ns	ns	ns	ns	0.42*∗*	0.41*∗*	0.44*∗*	ns	ns	ns	0.53*∗*	0.51*∗*	0.62*∗*	0.52*∗*	0.62*∗*	0.49*∗*
TW ER		ns	ns	ns	ns	ns	ns	0.39*∗*	0.43*∗*	0.53*∗*	ns	ns	ns	0.51*∗*	0.47*∗*	0.61*∗*	0.56*∗*	0.59*∗*	0.52*∗*
AP ER		ns	ns	ns	ns	ns	ns	0.48*∗*	0.39*∗*	0.52*∗*	ns	ns	ns	0.51*∗*	0.50*∗*	0.53*∗*	0.57*∗*	0.57*∗*	0.51*∗*
PT EL		ns	ns	ns	ns	ns	ns	0.41*∗*	0.44*∗*	0.39*∗*	0.39*∗*	ns	ns	0.52*∗*	0.48*∗*	0.56*∗*	0.58*∗*	0.63*∗*	0.63*∗*
TW EL		0.39*∗*	ns	ns	ns	ns	ns	0.39*∗*	0.46*∗*	ns	ns	0.40*∗*	ns	0.59*∗*	0.47*∗*	0.56*∗*	0.66*∗*	0.72*∗*	0.58*∗*
AP EL		ns	ns	ns	ns	ns	ns	0.42*∗*	0.48*∗*	ns	ns	ns	ns	0.52*∗*	ns	0.48*∗*	0.59*∗*	0.65*∗*	0.54*∗*

PT FR	KNEE	ns	ns	ns	ns	ns	ns	ns	ns	ns	ns	ns	ns	ns	ns	ns	0.63*∗*	0.65*∗*	0.72*∗*
TW FR		ns	ns	ns	ns	ns	ns	ns	0.38*∗*	0.40*∗*	ns	ns	ns	ns	ns	0.52*∗*	0.69*∗*	0.69*∗*	0.63*∗*
AP FR		ns	ns	ns	ns	ns	ns	ns	0.40*∗*	0.46*∗*	ns	ns	ns	0.47*∗*	ns	0.47*∗*	0.72*∗*	0.73*∗*	0.60*∗*
PT FL		ns	0.42*∗*	ns	ns	ns	ns	ns	0.51*∗*	ns	ns	0.46*∗*	ns	0.54*∗*	ns	0.52*∗*	0.68*∗*	0.60*∗*	0.53*∗*
TW FL		ns	0.46*∗*	ns	ns	ns	ns	0.42*∗*	0.41*∗*	ns	ns	0.54*∗*	ns	0.58*∗*	ns	0.60*∗*	0.67*∗*	0.68*∗*	0.46*∗*
AP FL		ns	0.45*∗*	ns	ns	ns	ns	0.45*∗*	0.43*∗*	ns	ns	0.46*∗*	ns	0.54*∗*	ns	0.60*∗*	0.63*∗*	0.65*∗*	0.45*∗*

PT ER	ANKLE	ns	0.45*∗*	-	ns	ns	-	0.39*∗*	ns	-	ns	ns	-	0.63*∗*	0.67*∗*	-	ns	0.43*∗*	-
TW ER		ns	ns	-	ns	ns	-	ns	ns	-	ns	ns	-	0.57*∗*	0.59*∗*	-	0.44*∗*	0.38*∗*	-
AP ER		ns	ns	-	ns	ns	-	ns	ns	-	ns	ns	-	0.61*∗*	0.54*∗*	-	0.39*∗*	ns	-
PT EL		ns	ns	-	ns	ns	-	0.58*∗*	0.57*∗*	-	ns	0.40*∗*	-	0.69*∗*	0.69*∗*	-	0.62*∗*	0.62*∗*	-
TW EL		ns	ns	-	ns	ns	-	0.54*∗*	0.55*∗*	-	ns	ns	-	0.47*∗*	0.52*∗*	-	0.63*∗*	0.57*∗*	-
AP EL		ns	ns	-	ns	ns	-	0.57*∗*	0.59*∗*	-	ns	ns	-	0.57*∗*	0.53*∗*	-	0.60*∗*	0.60*∗*	-

PT FR	ANKLE	ns	ns	-	ns	ns	-	ns	ns	-	ns	ns	-	0.67*∗*	0.64*∗*	-	ns	ns	-
TW FR		ns	ns	-	ns	ns	-	ns	ns	-	ns	ns	-	ns	ns	-	ns	ns	-
AP FR		ns	ns	-	ns	ns	-	ns	ns	-	ns	ns	-	0.51*∗*	0.48*∗*	-	ns	ns	-
PT FL		ns	ns	-	ns	ns	-	ns	ns	-	ns	ns	-	0.54*∗*	ns	-	0.40*∗*	ns	-
TW FL		ns	ns	-	ns	ns	-	ns	ns	-	ns	ns	-	ns	ns	-	0.51*∗*	0.42*∗*	-
AP FL		ns	ns	-	ns	ns	-	ns	ns	-	ns	ns	-	0.46*∗*	ns	-	ns	ns	-

*∗* p <0.05; ns: results statistical not significant; -: no measurement.

## Data Availability

The data (force-velocity parameters and walking parameters) used to support the findings of this study have been deposited in the WROVASC repository (e-mail: zuk@wssk.wroc.pl).
